# A reduced mechanical model for cAMP-modulated gating in HCN channels

**DOI:** 10.1038/srep40168

**Published:** 2017-01-11

**Authors:** Stephanie Weißgraeber, Andrea Saponaro, Gerhard Thiel, Kay Hamacher

**Affiliations:** 1 Department of Biology, TU Darmstadt, Germany; 2 Dept. of Biosciences, University of Milan, 20133 Milan, Italy

## Abstract

We developed an *in silico* mechanical model to analyze the process of cAMP-induced conformational modulations in hyperpolarization-activated cyclic nucleotide-gated (HCN) channels, which conduct cations across the membrane of mammalian heart and brain cells. The structural analysis reveals a quaternary twist in the cytosolic parts of the four subunits in the channel tetramer. This motion augments the intrinsic dynamics of the very same protein structure. The pronounced differences between the cAMP bound and unbound form include a mutual interaction between the C-linker of the cyclic nucleotide binding domain (CNBD) and the linker between the S4 and S5 transmembrane domain of the channel. This allows a mechanistic annotation of the twisting motion in relation to the allosteric modulation of voltage-dependent gating of this channel by cAMP.

Hyperpolarization-activated cyclic nucleotide-gated cation (HCN) channels conduct the “funny current” (*I*_*f*_), which determines pace-making in the heart and repetitive firing in neurons^1^. These channels, which are present in four isoforms HCN1–4 in humans, are members of the superfamily of voltage-gated K^+^ channels^2^.

Typical for these channels is the characteristic architecture with six transmembrane domains (TMDs) of which the 4th TMD contains the voltage sensor and the last two TMDs the pore^3^. At their cytosolic carboxyl termini, HCN channels have a canonical cyclic nucleotide-binding domain (CNBD), which is structurally similar to regulatory domains in different forms of life ranging from bacteria to humans^4^. HCN channels are inward rectifiers, which means that the open probability of the channel increases in response to hyperpolarization. This voltage-dependent activation of HCN channels is furthermore modulated by cyclic nucleotides. Upon binding of cAMP to the CNBD the opening of the channel occurs already at lower (negative) potentials^4^. The physiological effect of a rise in the cytosolic concentration of the signaling molecules is an acceleration of pace making^5^.

A challenge for unraveling the allosteric nature of channel regulation by both voltage and ligands is to understand how the two regulatory signals are processed in the context of the whole protein. One important component in this scenario, the CNBD, has been well studied. High resolution crystal structures are available for most HCN isoforms in the presence of cAMP^6^,^7^.

The CNBD consists of an eight-stranded β-roll, which is connected to an α-helix on the N-terminal side. Further downstream are two additional helices, which are termed B- and C-helices. The cAMP binding pocket is located in the β-roll and – when occupied by a cyclic nucleotide – it makes contact with the C-helix via the short P-helix. An important structure for the transmission of conformational information in the CNBD is the C-linker (CL). This helical domain links the CNBD to the channel pore. Information on the conformational dynamics in the CNBD between its bound and unbound form was recently provided by an NMR structure of a domain comprising CNBD and D′-F′ helices without cyclic nucleotide bound^8^. A comparison between the two structures now opens the possibility for understanding the conformational changes, which occur between binding and unbinding in the CNBD. The comparative analysis implies that cAMP binding generates large rigid body movements of the helical domains together with a stabilization of several of the helices.

In contrast to this detailed knowledge of the cytosolic domain, the structure and dynamics of the transmembrane region of HCN channels remain unknown up to now. In the absence of full structural knowledge of the entire channel it is challenging to understand the consequences of conformational changes in the CNBD on the transmembrane part of the protein and, in particular, on the gating of the pore.

In this study, we created a structural model of the HCN channel and simulated cAMP removal from the binding pocket using a coarse-grained approach to reveal the dynamics of the allosteric change upon nucleotide binding in the CNBD. We combined an elastic network model^9^,^10^ with Linear Response Theory^11^ to simulate opening of the cAMP binding pocket and analyze the mechanical response of the channel structure.

This methodological approach has several advantages in comparison to a full atomistic approach like traditional molecular dynamics. Foremost, research in the past years has shown that almost all allosteric conformational changes can be described by one or a subset of low frequency modes if the motion is highly collective, i.e., involves a high percentage of residues. Notable studies in the field were conducted by Xu *et al*., who were able to describe the conformational change between the tense and relaxed forms of hemoglobin using an elastic network model^12^, Wang *et al*., who analyzed the ratchet-like motion of the 70 S ribosome^13^ and many others^14^,^15^,^16^. Therefore – despite the lack of very detailed structural knowledge on the transmembrane parts – we can apply these coarse-grained models to homology structures. Furthermore, elastic network models are computationally inexpensive and robust to variations in adjustable parameters^17^,^18^. This property allowed us to take an orthogonal approach to traditional molecular dynamics studies: instead of gaining insight by detailed simulations of one particular aspect, we are able to compare observed effects in comparison to a large collection of perturbed scenarios and thus are able to identify unique aspects that distinguish, e.g., particular motions from the general dynamics– an approach well suited to understand allosteric conformational changes.

## Results

In order to understand the conformational changes in the CNBD following cAMP release from the binding pocket and the impact of this on the transmembrane part of the channel we constructed a homology model and applied a computational model of coarse-grained molecular dynamics on it (see Methods section for details). Details on the homology model, which is based on joining the crystal structures of the CNBD of HCN4 with the transmembrane part of the Kv1.2 channel as well as other details and quality assurance steps are shown in the supplementary material.

### Global Conformational Change Upon Ligand Dissociation

**Figure 1:**
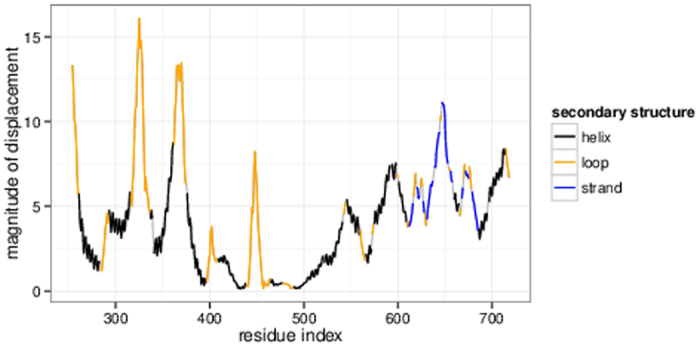
Magnitude of displacement of the C^*α*^ atoms of one HCN subunit after LRT. The *y*-axis is in arbitrary units.

Figure 1 shows the displacement of one subunit that occurs upon perturbation by an “external force”; the latter is effectively mimicking the (un)binding of cAMP and the induced forces. Because of the fourfold symmetry of the tetramer, the displacements of the respective residues in all subunits are equivalent.

In this plot residues 254 to 520 correspond to the transmembrane part of the channel followed by the C-terminal domain (residues 521 to 718). We emphasis that LRT is a qualitative rather than a quantitative assessment of protein dynamics. Hence, the approach provides information on the overall trend for the movement and the displacement of protein parts relative to each other, while the total magnitude of these displacements cannot be evaluated, i.e., the *y*-axis is in arbitrary units.

The data in Fig. 1 show that the pore and filter region with the conserved CIGYG motif (residues 478 to 482) undergo almost no displacement following unbinding of cAMP.

**Figure 2:**
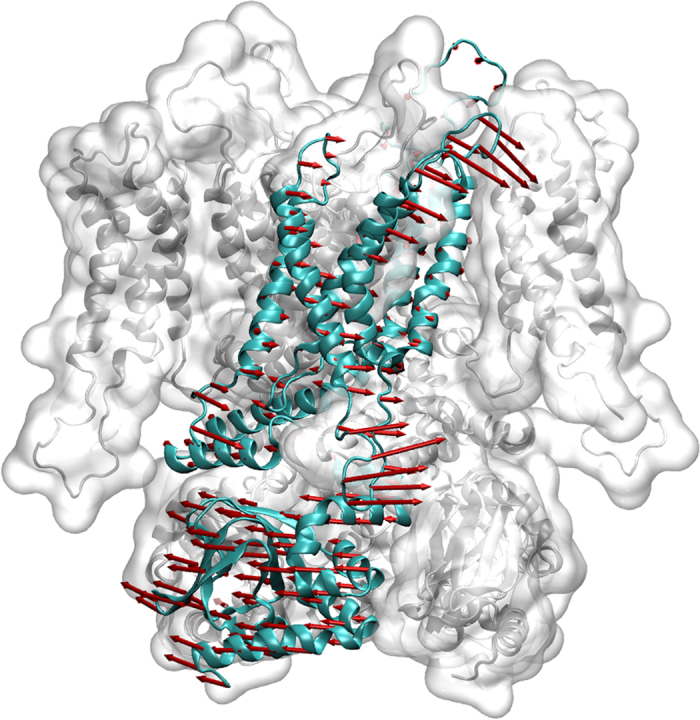
HCN4 tetramer, one chain is shown in turquoise in a cartoon representation. Arrows represent the displacement after LRT force application to the cAMP binding pocket residues of all four subunits simultaneously.

The overall motion of the HCN4 protein is shown in Fig. 2: arrows indicate the direction and their length the magnitude of displacement after LRT. Again, interpretation of arrow lengths can only be done by ratios, as the LRT method is not quantitatively predictive. The data in Fig. 2 show that application of force to the six residues of the cAMP binding pocket leads to distinct short- and long range movements in the protein. Most striking is a torsion of the CNBD against the transmembrane domain. The relative conformation of the outer region of the pore (pore helix and filter region) is maintained during LRT.[Fig f3]

Such a torsion of the transmembrane domain against the non-membrane region has been observed for several other channels: the closely related cyclic nucleotide-modulated, bacterial MlotiK1 (via cryo-electron microscopy of the open and closed state)^19^, the mechanosensitive MscL channel of *Mycobacterium tuberculosis* (by comparing crystal structures of open and closed state)^20^, and the nicotinic acetylcholine receptor (ENM analysis)^21^.

Furthermore, the opening mechanism of several potassium channels has been shown to be a quaternary twist^22^,^23^. Alam *et al*.^24^ were able to create a high resolution crystal structure for NaK in the open state and reported a torsion for this sodium and potassium conducting channel compared to the crystallized channel in the closed state^25^. They also discovered that the conformation of the filter region of the pore is almost the same in both states, which is in line with observations for the potassium channels KcsA (crystal structure in the closed state^26^) and MthK (crystal structure in the open state^27^). Thus, our observation of a quaternary torsion agrees with previous findings in other ion channels in relation to gating.

A closer look at the interface between the transmembrane part of the channel and its C-terminal region reveals that the torsion of the domains moves the S4–S5 - and the C-linker toward each other and enables closer interaction of these two structural elements. Hence, cAMP removal from its binding pocket brings the S4–S5-linker closer to the inhibiting CNBD. This finding is consistent with experimental data showing that the S4S5- and the C-linker move in HCN channels relative to each other during gating^28^. Our results now imply that this process is also involved in the channel’s reaction to cAMP binding. This agrees well with previous findings on the influence of the transmembrane voltage sensor domain on the CNBD^29^,^30^.

While it has not been revealed up to this point, how cAMP modulates channel gating exactly, we do know that a complete removal of the CNBD has an effect similar to cAMP binding: after truncation of the CNBD the channel opens already at less negative voltages, i.e., the CNBD in the ligand-free state inhibits channel opening^31^.

The S4–S5-linker transmits the reaction of the voltage sensor to the pore-forming parts of the channel. Studies have suggested that an interaction of the C-linker with the S4–S5-linker couples voltage-gating and allosteric modulation of the channel by cAMP.

If cAMP removal brings the C-linkers closer to the S4–S5-linkers of their neighboring subunits, this in turn means that cAMP binding moves them further away thereby preventing interactions. This mechanism could be part of the reason why cAMP binding revokes the inhibitory influence of the CNBD.

### Comparison of 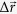 with Low Frequency Modes

We computed the overlap 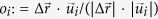 between the non-degenerate eigenvectors 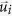 and the displacement vector 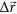 from LRT. The detailed results are shown in the supplemental material (Fig. 4 and and Sec. 4 therein). Eigenmode no. 8 – a soft mode – showed the highest overlap to 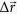 with *o*_8_ = 0.73. Thus, non-surprisingly, the movement of the channel upon cAMP (un)binding corresponds to a high degree with a “soft mode” and thus a functional movement. “Soft modes” are those movements in an elastic network model with a higher degree of cooperativity of residues and accompanying slower velocities^32^ and thus lesser energy to be spend to evoke it.

Some movements of proteins are composed of several superimposed modes^33^. To clarify whether this is the case for cAMP dissociation in our model, linear combinations of the eigenvectors that featured the highest overlap with 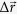 were computed.

**Figure 3 f3:**
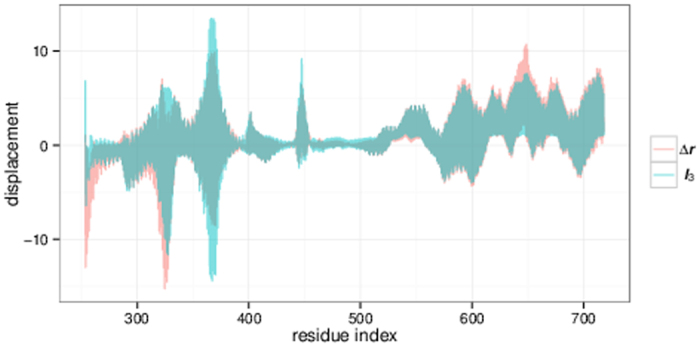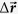 **from LRT compared to a linear combination of three eigenvectors (**
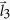
**, cyan) with the highest overlap with** 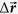 **(red): no. 8, no. 13 and no. 10 (overlap: 0.95).** Eigenvectors were derived from a singular value decomposition of the Hessian matrix of the system. Plotted vectors were scaled to the same magnitude for better comparability.

Figure 5b of the supplemental material combination of two eigenvectors (no. 8 and no. 13), which increased the overlap to 0.85 in comparison to the sole eigenvector no. 8 (show for comparison in Fig. 5a in the supplemental material). The overlap between 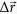 and the linear combination of three eigenvectors (no. 8, no. 13 and no. 10) was 0.95. It is illustrated in Fig. 3. Adding a fourth eigenvector only slightly increases the overlap to 0.97. Thus, three eigenvectors are sufficient to describe the displacement upon force application to the cAMP binding pocket almost perfectly – and they are all functional, soft modes. This implies that our model relates the effect of cAMP (un)binding solely to functional, global movements and not to localized modes that are known to involve only a few residues each.

Congruence of the eigenmodes (especially the linear combination of three modes) and 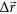 is best in the C-terminal domain and the helical regions of the transmembrane domain. The largest divergence is found for the loops in the transmembrane domain. As mentioned in the evaluation of the homology model, modeled loop regions are most likely to differ from the native state of the protein, which might be the reason for this discrepancy. Nevertheless, the fact that we only need three eigenvectors for almost perfect overlaps shows that the allosteric conformational change we discovered using LRT is an intrinsic property of the HCN4 channel structure; if it were not for this fact, the composition of the movement would necessarily involve many and not just three modes.

### Synergies in Global Conformational Change Upon Ligand Dissociation and Voltage

**Figure 4:**
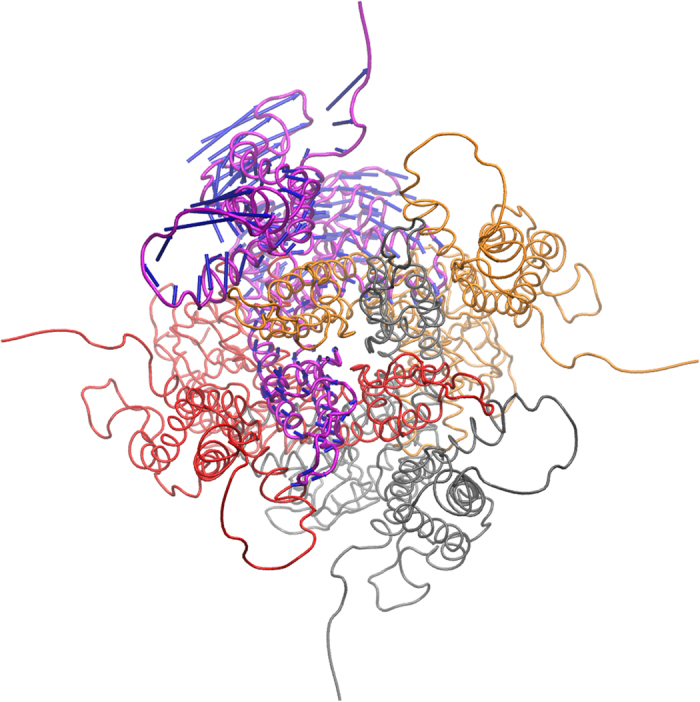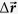 **per residue (blue arrows) in one monomer (purple) from LRT upon an external electrical field across the membrane.**

As is well-known cAMP-binding reduces the necessary external voltage for channel opening^4^. We performed additional ENM experiments to study how this effect can mechanically explained. To this end we applied an additional force to the charged residues in the channel’s tetramer. We then computed the mechanical response within the LRT approximation to this external perturbation. We found that the structural shift in the residue positions is very similar to the ones obtained to sole cAMP binding – effectively a twist-like motion. This is illustrated in Fig. 4.

The magnitude and direction of this shift 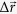 is comparable to the one upon binding the cAMP. This is shown in the supplemental material in its Sec. 6.

It is well-known that the removal of the CNBD has similar effects as cAMP binding^34^. While this would be an interesting control for a theoretical model, we cannot follow this route in our present setup due to inherent limitations of the method: cAMP is here not considered as a “particle”; its absence or presence is solely modeled via a perturbing force in Eq. 3. Now, the removal of the entire CNBD is a much larger perturbation and implies the removal of a collection of “particles” from the Hamiltonian H itself. From this we cannot directly compute a positional shift in 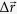.

### Identifying Key Residues in Channel Modulation

3,270 contact switch-offs were performed and force was applied to open the cAMP binding pocket. The impact was analyzed by comparing the displacement vector of each altered system with one switched off contact to the original 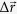.

**Figure 5:**
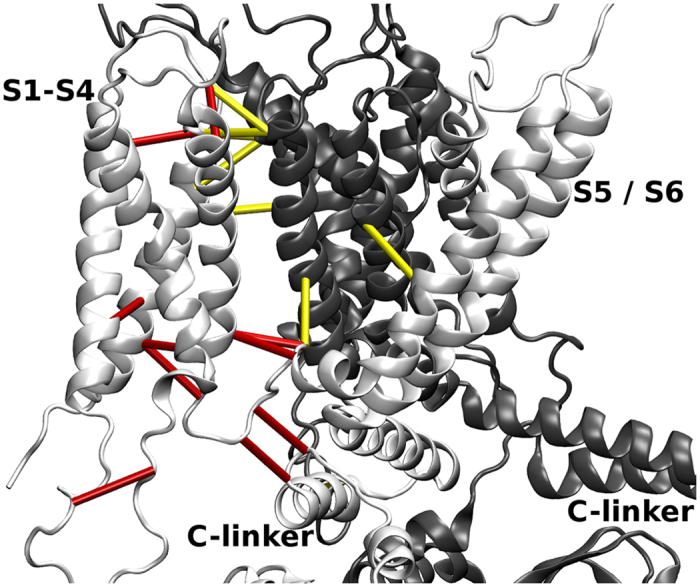
Transmembrane region and part of the C-linker of two neighboring subunits of HCN4 (white and gray). The contacts that cause significant change of displacement when switched off are indicated by cylinders. Intra-subunit contacts are drawn in red (282–295, 269–309, 322–334, 286–381, 266–396, 266–399, 395–407, 395–408, 402–560, 403–561), inter-subunit contacts in yellow (430–277, 466–278, 466–282, 438–284, 438–286, 424–407, 497–422, 331–545).

**Figure 6:**
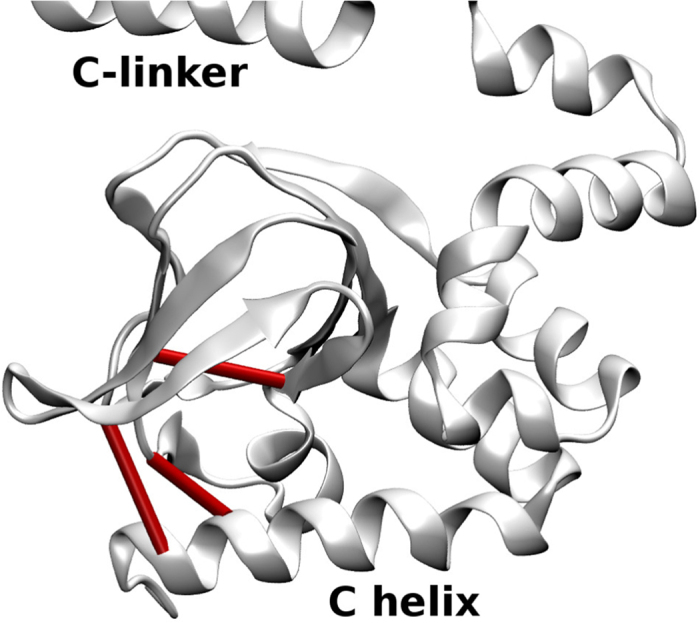
C-terminal domain with part of the C-linker and CNBD. The contacts that cause significant change of displacement when switched off are indicated by cylinders (642–658, 670–710, 649–713). Colors as in Fig. 5.

Since we wanted to detect contacts important for global conformational rearrangement, we focused on switch-offs that induced a significant change of displacement (>0.1) for at least 15 residues of each subunit. This was the case for 21 contacts. The change in magnitude of displacement after LRT that they caused is illustrated in the Supplemental Material (Figure 3) and their location in the protein is indicated in Figs 5 and 6.

As is evident from the upper six panels in Supplemental Figure 3 the four-helix bundle S1–S4 is stabilized. This is the reason why the interruption of these contacts induces the largest change in the N-terminal part of the subunit. The contacts 395–407 and 395–408 connect the four-helix bundle with the inner region of the transmembrane domain across secondary structure elements. Both, residue 407 and 408, are highly conserved, which (partially) illustrates the selective pressure. The exception is interaction 322–334: both of these residues lie in the same loop but on opposite sites, thereby influencing the adjoining helices. This shows that interactions between the transmembrane helices play an important role in the allosteric reaction of the channel to cAMP binding and dissociation. Both intra- and inter-subunit contacts are involved in this process.

## Discussion

We built a homology model of the HCN4 channel transmembrane region and modeled it in conjunction with the available crystal structure of the C-terminal domain. The joined model was used to study allosteric conformational change associated with cAMP release. To this end, an elastic network model of the HCN tetramer was constructed. cAMP dissociation was simulated by a force that opened the binding pocket. The resulting conformational change of the HCN tetramer was compared to low frequency modes of the ENM. In addition, we conducted a switch-off screening to identify key residues in the process. Up to now, no such detailed computational analysis of cAMP modulation in HCN has been published.

cAMP binding removes the inhibitory effect of the CNBD and thereby reduces the hyperpolarization threshold that needs to be reached for channel gating. Our results suggest that the quaternary twist, which has been shown to be the opening mechanism for several ion channels, is already part of the allosteric reaction of the channel upon cAMP binding.

We could also show that interaction between the S4–S5-linker in the transmembrane domain and the C-linker is influenced by the allosteric rearrangement. This might be part of the mechanism of how cAMP modulates channel behavior. As the group around Sanguinetti found out, the S4–S5-linker participates in channel gating^35^. They also presented mutational studies which suggested an interaction between the S4–S5-linker and the C-linker^36^. Our results point in a similar direction: The S4–S5-linker seems to be involved not only in channel gating but also in cAMP modulation – a fact also recently indicated by double electron-electron resonance experiments that pointed to torison-like movements upon linker length changes^37^.

The search for key players in cAMP-induced allosteric conformational change revealed that the most important contacts are those between the helices of the transmembrane domain. Here, both, intra- and inter-subunit contacts between the transmembrane areas are relevant in this process; thus, the effect can only be observed in the cooperatively acting, tetrameric structure and not solely a monomer.

Since HCN1, HCN2 and HCN4 are very similar in sequence and structure and the methods applied in this study are coarse-grained and insensitive against sequence variation, the insight gained for HCN4 most likely holds for HCN1 and HCN2 as well.

## Methods

### Homology Model of HCN4

For our structural investigation we developed a homology model of the HCN4 tetramer and the CNBD. Details are discussed in the supplemental material.

### Anisotropic Network Model

Elastic network models (ENMs) are a coarse-grained approach to study protein dynamics. They map the protein and its full, classical potential to a mass-and-spring network, where each amino acid is a node and the springs are the interactions between them^38^,^39^. The coordinates of the nodes in three-dimensional space are the coordinates of their C^*α*^ atoms 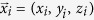 with 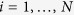, *N* being the number of nodes.

The anisotropic network model (ANM)^9^,^10^,^40^ is a special type of elastic network model. The potential *V* is given as follows:


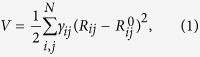


where the sum runs over all contacts, e.g., residue pairs *i* and *j* whose Euclidean distance is smaller than some threshold. *γ*_*ij*_ is the spring constant for the interaction between residues *i* and *j*, which can be interaction type-specific; 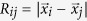 is the respective distance; and 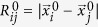 the distance of these residues in the equilibrium state, i.e., the starting structure^41^.

The Hessian matrix 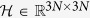 of the system is the second derivative of the potential with respect to the 3 *N* cartesian coordinates.

Singular value decomposition yields the eigenvalues and eigenvectors of the Hessian 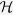
42. Six of the Hessian’s eigenvalues vanish due to three rotational and three translational degrees of freedom. From the 3 *N − *6 non-vanishing eigenvalues *λ*_*k*_ and their corresponding eigenvectors 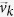 the Moore-Penrose pseudoinverse 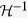 of the Hessian can be computed^43^,^44^:


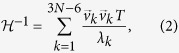


resulting in the mechanical covariance matrix of the ANM, which contains information about how the residues in the elastic network are coupled dynamically in an equilibrium state.

The eigenvectors of the Hessian matrix 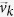 of an ANM correspond to the fluctuations a protein undergoes. Each of these eigenvectors contains 3 *N* entries since the motion of each residue in all three spatial dimensions is required to describe the complete oscillation of the protein. Their respective eigenvalues are the square of the frequencies of these fluctuations. High frequency fluctuations are local, stabilizing movements, while low frequency modes (also called soft modes) describe global, collective motions, that affect large parts of the protein^32^.

Several studies in the past have shown that low frequency modes obtained by ENM analysis provide valuable insight into protein mechanics^45^. According to Tobi *et al*., ligand binding does not induce conformational change but stabilizes conformations that are already accessible to the ligand-free form of the protein^46^. Therefore, the native state can be employed to gather information about allosteric reaction to ligand binding.

For our HCN4 homology model, we built an ANM using the BioPhysConnectoR package^47^ in R. The contact cutoff was set to 10 Å covalent bond strengths were set to 82*RT*/Å^2^ with *R* = *k*_*B*_*N*_*A*_ (*k*_*B*_: Boltzmann constant; *N*_*A*_: Avogadro constant; *T*: temperature)^10^. Non-covalent interactions were set to 3.166*RT*/Å^2^, which is the average value of non-covalent interactions in the Miyazawa-Jernigan matrix^48^.

This ANM Hessian depends on the HCN4 contact map – the binary matrix representing contacts within the above mentioned contact cutoff. Obviously, structural inaccuracies that can be expected in any homology model lead to changes in the contact map. It is known, however, that ENMs and ANMs in particular are robust against such “homology model noise”^49^.

### Linear Response Theory

In 2005, Ikeguchi *et al*. used linear response theory (LRT)^50^ to simulate the binding of ligands to proteins^11^. LRT states that the response to a perturbation due to ligand binding is related to the equilibrium fluctuations of the receptor in the unperturbed state.

The expected coordinate shift for all residues 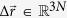 can be computed from the covariance matrix 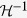 of the ligand-free state and the perturbation upon ligand binding, which is simply a force acting on the binding pocket:


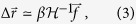


with *β* being the force constant and 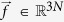 the vector whose components are the forces acting upon each residue in the three spatial directions.

In this study, a force was simultaneously applied to six residues which constitute the cAMP binding pocket^51^ (V642, T644, K648, E660, R669, R710) to mimic the effects of cAMP binding. The direction of the force was chosen to point from the geometric center of the heavy atoms of the bound cAMP (extracted from the PDB data) towards the C^*α*^ atom of the forced residue. As atomistic – up to quantum-mechanical – details of the binding process remain illusive, all force vectors were normalized to the same length so that a force of the same strength was applied to each binding pocket residue. This restricts us to qualitative insight only. Still, in the subsequent parts of this study we can use “coarse-grained insight” for an assessment of the binding process and its implications for the conformational changes.

### Low Frequency Modes as Reference Motions

Eigenmodes which introduce the same change in all four subunits are considered non-degenerate. They were shown to describe cooperative transitions of multimeric proteins well^52^. Therefore, our analysis was restricted to these non-degenerate modes. To perform the symmetry check the magnitude of each three-dimensional displacement vector 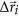 (as part of 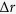 in Eq. 3) was computed. For the resulting *N*-dimensional vector of magnitudes, Pearson’s correlation coefficient was computed between the first, second, third and fourth quarter. Modes were considered non-degenerate if the correlation between all quarters was higher than 0.95.

In a next step, the overlap between each non-degenerate eigenvector and the displacement vector 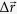 from LRT in Eq. 3 was computed. The overlap *I* between two vectors 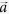 and 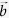 is


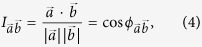


where 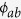 is the angle between 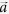 and 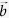
53.

We also computed linear combinations 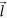 of *k* non-degenerate modes 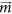 that best described the displacement upon LRT, i.e., had the highest overlap with 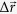:


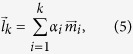


with 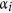 being the scaling factor for the *i*th mode that is calculated as follows: Since the eigenvectors of the Hessian form an orthonormal basis, we can determine the part of 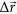 that can be described through mode 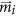 by normalizing 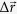 (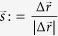) and projecting 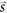 onto 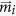
54:


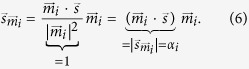


Thus, the scaling factor 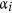 is the dot product of 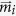 and 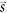, which also corresponds to the overlap between 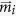 and 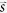:


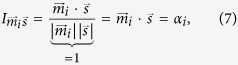


The overlap of these linear combinations 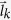 with 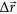 was then computed.

### Assessment of Residue Contacts Using Switch-Off Analysis

In order to identify interactions important for the response of the protein to ligand binding, we performed a gedankenexperiment: all contacts are switched off one by one, i.e., the interaction strength 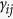 for the respective amino acid pair is set to zero in Eq. 1 before applying the force that mimics the effect of ligand binding. This method is somewhat similar to an alanine scan in the laboratory with two modifications: first, an alanine mutation only reduces interaction strength^48^, it does not completely remove it. Second, mutation influences all interactions of a residue at once, while our switch-off model allows investigation of *individual* interactions between two residues^10^,^55^. To maintain symmetry in the tetramer all corresponding contacts were switched off in all subunits simultaneously.

## Additional Information

**How to cite this article**: Weißgraeber, S. *et al*. A reduced mechanical model for cAMP-modulated gating in HCN channels. *Sci. Rep.* **7**, 40168; doi: 10.1038/srep40168 (2017).

**Publisher's note:** Springer Nature remains neutral with regard to jurisdictional claims in published maps and institutional affiliations.

## Supplementary Material

Supplementary Material

## References

[b1] Barbuti A. . Molecular composition and functional properties of f-channels in murine embryonic stem cell-derived pacemaker cells. Journal of Molecular and Cellular Cardiology 46, 343–351 (2009). 19135060 10.1016/j.yjmcc.2008.12.001

[b2] Accili E. A., Proenza C., Baruscotti M. & DiFrancesco D. From funny current to hcn channels: 20 years of excitation. Physiology 17, 32–37 (2002). 10.1152/physiologyonline.2002.17.1.32 11821534

[b3] Biel M., Wahl-Schott C., Michalakis S. & Zong X. Hyperpolarization-activated cation channels: from genes to function. Physiological reviews 89, 847–885 (2009). 19584315 10.1152/physrev.00029.2008

[b4] Craven K. B. & Zagotta W. N. Cng and hcn channels: two peas, one pod. Annu. Rev. Physiol. 68, 375–401 (2006). 16460277 10.1146/annurev.physiol.68.040104.134728

[b5] Baruscotti M., Bottelli G., Milanesi R., DiFrancesco J. C. & DiFrancesco D. Hcn-related channelopathies. Pflügers Archiv-European Journal of Physiology 460, 405–415 (2010). 20213494 10.1007/s00424-010-0810-8

[b6] Lolicato M. . Tetramerization dynamics of c-terminal domain underlies isoform-specific camp gating in hyperpolarization-activated cyclic nucleotide-gated channels. Journal of Biological Chemistry 286, 44811–44820 (2011). 22006928 10.1074/jbc.M111.297606 PMC3247997

[b7] Zagotta W. N. . Structural basis for modulation and agonist specificity of HCN pacemaker channels. Nature 425, 200–205 (2003). 12968185 10.1038/nature01922

[b8] Saponaro A. . Structural basis for the mutual antagonism of cAMP and TRIP8b in regulating HCN channel function. Proceedings of the National Academy of Sciences 111, 14577–14582 (2014). 10.1073/pnas.1410389111 PMC4210022 25197093

[b9] Atilgan A. R. . Anisotropy of fluctuation dynamics of proteins with an elastic network model. Biophysical Journal 80, 505–515 (2001). 11159421 10.1016/S0006-3495(01)76033-X PMC1301252

[b10] Hamacher K. & McCammon J. A. Computing the amino acid specificity of fluctuations in biomolecular systems. Journal of Chemical Theory and Computation 2, 873–878 (2006). 26626694 10.1021/ct050247s

[b11] Ikeguchi M., Ueno J., Sato M. & Kidera A. Protein structural change upon ligand binding: linear response theory. Physical Review Letters 94, 078102 (2005). 15783858 10.1103/PhysRevLett.94.078102

[b12] Xu C., Tobi D. & Bahar I. Allosteric changes in protein structure computed by a simple mechanical model: hemoglobin T <−> R2 transition. Journal of Molecular Biology 333, 153–168 (2003). 14516750 10.1016/j.jmb.2003.08.027

[b13] Wang Y., Rader A., Bahar I. & Jernigan R. L. Global ribosome motions revealed with elastic network model. Journal of Structural Biology 147, 302–314 (2004). 15450299 10.1016/j.jsb.2004.01.005

[b14] Tama F. & Sanejouand Y. H. Conformational change of proteins arising from normal mode calculations. Protein Engineering 14, 1–6 (2001). 11287673 10.1093/protein/14.1.1

[b15] Yang L., Song G. & Jernigan R. L. How well can we understand large-scale protein motions using normal modes of elastic network models? Biophysical Journal 93, 920–929 (2007). 17483178 10.1529/biophysj.106.095927 PMC1913142

[b16] Bahar I., Lezon T. R., Yang L.-W. & Eyal E. Global dynamics of proteins: bridging between structure and function. Annual Review of Biophysics 39, 23–42 (2010). 10.1146/annurev.biophys.093008.131258 PMC2938190 20192781

[b17] Doruker P., Jernigan R. L. & Bahar I. Dynamics of large proteins through hierarchical levels of coarse-grained structures. Journal of Computational Chemistry 23, 119–127 (2002). 11913377 10.1002/jcc.1160

[b18] Lu M. & Ma J. The role of shape in determining molecular motions. Biophysical Journal 89, 2395–2401 (2005). 16055547 10.1529/biophysj.105.065904 PMC1366739

[b19] Kowal J. . Ligand-induced structural changes in the cyclic nucleotide-modulated potassium channel MloK1. Nature Communications 5 (2014). 10.1038/ncomms4106 PMC4086158 24469021

[b20] Sukharev S., Durell S. R. & Guy H. R. Structural models of the MscL gating mechanism. Biophysical Journal 81, 917–936 (2001). 11463635 10.1016/S0006-3495(01)75751-7 PMC1301563

[b21] Szarecka A., Xu Y. & Tang P. Dynamics of heteropentameric nicotinic acetylcholine receptor: implications of the gating mechanism. Proteins: Structure, Function, and Bioinformatics 68, 948–960 (2007). 10.1002/prot.21462 17546671

[b22] Shrivastava I. H. & Bahar I. Common mechanism of pore opening shared by five different potassium channels. Biophysical Journal 90, 3929–3940 (2006). 16533848 10.1529/biophysj.105.080093 PMC1459499

[b23] Haliloglu T. & Ben-Tal N. Cooperative transition between open and closed conformations in potassium channels. PLoS Computational Biology 4, e1000164 (2008). 18769593 10.1371/journal.pcbi.1000164 PMC2528004

[b24] Alam A. & Jiang Y. High-resolution structure of the open NaK channel. Nature Structural & Molecular Biology 16, 30–34 (2009). 10.1038/nsmb.1531 PMC2615073 19098917

[b25] Shi N., Ye S., Alam A., Chen L. & Jiang Y. Atomic structure of a Na^+^-and K^+^-conducting channel. Nature 440, 570–574 (2006). 16467789 10.1038/nature04508

[b26] Zhou Y., Morais-Cabral J. H., Kaufman A. & MacKinnon R. Chemistry of ion coordination and hydration revealed by a K^+^; channel-Fab complex at 2.0 Å resolution. Nature 414, 43–48 (2001). 11689936 10.1038/35102009

[b27] Jiang Y. . Crystal structure and mechanism of a calcium-gated potassium channel. Nature 417, 515–522 (2002). 12037559 10.1038/417515a

[b28] Kwan D. C., Prole D. L. & Yellen G. Structural changes during HCN channel gating defined by high affinity metal bridges. The Journal of General Physiology 140, 279–291 (2012). 22930802 10.1085/jgp.201210838 PMC3434101

[b29] Kusch J. . Interdependence of receptor activation and ligand binding in HCN2 pacemaker channels. Neuron 67, 75–85 (2010). 20624593 10.1016/j.neuron.2010.05.022

[b30] Thon S., Schulz E., Kusch J. & Benndorf K. Conformational flip of nonactivated HCN2 channel subunits evoked by cyclic nucleotides. Biophys. J. 109, 2268–76 (2015). 26636938 10.1016/j.bpj.2015.08.054 PMC4675818

[b31] Wainger B. J., DeGennaro M., Santoro B., Siegelbaum S. A. & Tibbs G. R. Molecular mechanism of camp modulation of HCN pacemaker channels. Nature 411, 805–810 (2001). 11459060 10.1038/35081088

[b32] Bahar I., Lezon T. R., Bakan A. & Shrivastava I. H. Normal mode analysis of biomolecular structures: functional mechanisms of membrane proteins. Chemical Reviews 110, 1463–1497 (2010). 19785456 10.1021/cr900095e PMC2836427

[b33] Cui Q. & Bahar I. Normal mode analysis: theory and applications to biological and chemical systems (CRC press, 2005).

[b34] Wainger B. J. . Molecular mechanism of cAMP modulation of HCN pacemaker channels. Nature 411, 805–810 (2001). 11459060 10.1038/35081088

[b35] Chen J., Mitcheson J. S., Tristani-Firouzi M., Lin M. & Sanguinetti M. C. The S4–S5 linker couples voltage sensing and activation of pacemaker channels. Proceedings of the National Academy of Sciences of the United States of America 98, 11277–11282 (2001). 11553787 10.1073/pnas.201250598 PMC58720

[b36] Decher N., Chen J. & Sanguinetti M. C. Voltage-dependent gating of hyperpolarization-activated, cyclic nucleotide-gated pacemaker channels molecular coupling between the S4–S5 and C-linkers. Journal of Biological Chemistry 279, 13859–13865 (2004). 14726518 10.1074/jbc.M313704200

[b37] Puljung M. C., DeBerg H. A., Zagotta W. N. & Stoll S. Double electron – electron resonance reveals cAMP-induced conformational change in HCN channels. Proceedings of the National Academy of Sciences 111, 9816–9821 (2014). 10.1073/pnas.1405371111 PMC4103371 24958877

[b38] Tirion M. M. Large amplitude elastic motions in proteins from a single-parameter, atomic analysis. Physical Review Letters 77, 1905–1908 (1996). 10063201 10.1103/PhysRevLett.77.1905

[b39] Bahar I., Atilgan A. R. & Erman B. Direct evaluation of thermal fluctuations in proteins using a single-parameter harmonic potential. Folding and Design 2, 173–181 (1997). 9218955 10.1016/S1359-0278(97)00024-2

[b40] Hamacher K. Efficient quantification of the importance of contacts for the dynamical stability of proteins. J. Comp. Chem. 32, 810–815 (2011). 20957707 10.1002/jcc.21659

[b41] Hinsen K. Analysis of domain motions by approximate normal mode calculations. Proteins: Structure, Function, and Genetics 33, 417–429 (1998). 10.1002/(sici)1097-0134(19981115)33:3<417::aid-prot10>3.0.co;2-8 9829700

[b42] Golub G. & Kahan W. Calculating the singular values and pseudo-inverse of a matrix. SIAM Journal on Numerical Analysis 2, 205–224 (1965).

[b43] Moore E. On the reciprocal of the general algebraic matrix. Bulletin of the American Mathematical Society 26, 394–395 (1920).

[b44] Penrose R. A generalized inverse for matrices. In Mathematical Proceedings of the Cambridge Philosophical Society vol. 51, 406–413 (Cambridge University Press, 1955).

[b45] Bahar I., Chennubhotla C. & Tobi D. Intrinsic dynamics of enzymes in the unbound state and relation to allosteric regulation. Current Opinion in Structural Biology 17, 633–640 (2007). 18024008 10.1016/j.sbi.2007.09.011 PMC2197162

[b46] Tobi D. & Bahar I. Structural changes involved in protein binding correlate with intrinsic motions of proteins in the unbound state. Proceedings of the National Academy of Sciences of the United States of America 102, 18908–18913 (2005). 16354836 10.1073/pnas.0507603102 PMC1323175

[b47] Hoffgaard F., Weil P. & Hamacher K. BioPhysConnectoR: Connecting sequence information and biophysical models. BMC Bioinformatics 11, 199 (2010). 20412558 10.1186/1471-2105-11-199 PMC2868838

[b48] Miyazawa S. & Jernigan R. L. Estimation of effective interresidue contact energies from protein crystal structures: quasi-chemical approximation. Macromolecules 18, 534–552 (1985).

[b49] Hamacher K., Trylska J. & McCammon J. A. Dependency map of proteins in the small ribosomal subunit. PLoS Computational Biology 2, e10 (2006). 16485038 10.1371/journal.pcbi.0020010 PMC1364506

[b50] Hansen J. & McDonald I. Theory of Simple Liquids, 2nd (Academic, New York, 1986).

[b51] Altieri S. L. . Structural and energetic analysis of activation by a cyclic nucleotide binding domain. Journal of Molecular Biology 381, 655–669 (2008). 18619611 10.1016/j.jmb.2008.06.011 PMC2555981

[b52] Bahar I. On the functional significance of soft modes predicted by coarse-grained models for membrane proteins. The Journal of General Physiology 135, 563–573 (2010). 20513758 10.1085/jgp.200910368 PMC2888054

[b53] Marques O. & Sanejouand Y. H. Hinge-bending motion in citrate synthase arising from normal mode calculations. Proteins: Structure, Function, and Bioinformatics 23, 557–560 (1995). 10.1002/prot.340230410 8749851

[b54] Jänich K. Lineare Algebra (Springer, 2008).

[b55] Hamacher K. Relating sequence evolution of HIV1-protease to its underlying molecular mechanics. Gene 422, 30–36 (2008). 18590806 10.1016/j.gene.2008.06.007

