# Supplementary material for: A reduced mechanical model for cAMP-modulated gating in HCN channels
Source: Sci Rep. 2017 Jan 11;7:40168. doi: 10.1038/srep40168 (PMC5225470; doi:10.1038/srep40168)
Supplement: Supplementary Material [file srep40168-s1.pdf]

Supplemental material
"A reduced mechanical model for cAMP-modulated gating in HCN channels"

Stephanie Weißgraeber[1], Andrea Saponaro[2], Gerhard Thiel[1], and Kay Hamacher[1]
[1] Department of Biology, TU Darmstadt, Germany
[2] Dept. of Biosciences, University of Milan, 20133 Milan, Italy

October 31, 2016

1 Homology Model of HCN4

The homology model of the HCN4 channel transmembrane domain was constructed with `SWISS-MODEL` [1, 2]. The HCN4 sequence with GenBank [3] identifier 29840776 was chosen as a target sequence. The transmembrane domain of a related K_v channel (PDB [4] ID: 3LNM, chain B, biological assembly [5]) served as template. Sequence alignment and modeling were performed with `SWISS-MODEL`.

The loops between the transmembrane helices were remodeled *de novo* using the loop modeling function of `MODELLER` [6]. `STRIDE` [7] data were used to identify loop regions.

A symmetric tetramer was constructed by coordinate superposition over C^α atoms of the modeled monomer and each of the four subunits of the template. To improve assembly of the tetramer and avoid steric clashes, energy minimization was performed *in vacuo* with `GROMACS 4.5` [8] (force field: amber; integrator: steep; nsteps: 20 000; ns_type: grid; coulombtype: shift; rcoulomb: 1.3; rcoulomb_switch: 1.0; vdwtype: shift; rvdw: 1.3; rvdw_switch: 1.0; pbc: xyz; rlist: 2.0; optimize_fft: yes; emtol: 500 ; all parameters not listed were set to default values).

To obtain a complete model of the HCN4 channel, the homology model of the tetrameric transmembrane domain was joined to the crystal structure of the tetrameric C-terminal domain (PDB ID: 3U11 [9]). An overlap of three amino acids between the two tetramers was used to merge the chains by superposing the coordinates of the C^α atoms of residue 521 (the starting residue of the crystal structure of the C-terminal domain) and rotation/translation of the PDB file coordinates. Afterwards, the energy of the joined model was again minimized *in vacuo* with `GROMACS 4.5`.

2 Evaluation of the HCN4 Model

At present, there is no crystal structure data for the transmembrane region of an HCN channel. Therefore, a homology model was constructed with `SWISS-MODEL` using the transmembrane domain of a related K_v channel as template.

Several modeling attempts using three different homology modeling programs (`SWISS-MODEL`, `I-TASSER`, `MODELLER`) and four templates (PDB IDs: 3LNM, 2R9R, 3BEH, 3LUT) were compared. To this end, the pairwise sequence alignments of HCN4 and related K_v channels were inspected. The best alignment of HCN4 could be achieved with the template 3LNM.

The regions of the HCN4 sequence that were annotated as transmembrane helices and pore loop in GenBank were well aligned with the corresponding structural elements of 3LNM. For this reason and because of the fact that the structure of the six transmembrane helices should be fairly conserved, 3LNM was chosen as template sequence, in spite of low (11%) sequence identity to HCN4.

`SWISS-MODEL` accomplished the best alignment of HCN4 with the template 3LNM as well as the best homology model concerning structural integrity, secondary structure elements matching sequence annotation by GenBank and QMEAN [10] scores. Therefore, this approach was used to create the final model.

The homology model of the transmembrane region was added to the crystal structure of the C-terminal domain (PDB ID 3U11) to obtain a model including residues 254 to 718 of the functional channel comprising the full transmembrane domain, the C-Linker and the cyclic nucleotide-binding domain (CNBD).

Figure 1 shows the assembled tetramer: side view with the transmembrane region in the upper part in 1a and top view from the extracellular space in 1b. A single subunit is depicted in Figure 1c and an annotated illustration of the homology modeled transmembrane domain can be found in Figure 1d.

The QMEAN server was used to evaluate the joined model. Figure 2 shows the estimated error per residue of the HCN4 joined model. Unfortunately, the QMEAN score is not a completely reliable measure for judging the quality of membrane protein models, since the data set on which its computation is based mostly consists of soluble proteins [11].

Nevertheless, the model is qualified for our coarse-grained approach as most parts are within an error range of less than 3.5 Å. However, we need to keep in mind that results for the loop regions should be treated with caution.

3 Modeling the Release of cAMP via Force Application

The model for obtaining the cAMP-free conformation of HCN was based on the MlotiK1 and CAP cAMP-binding domains as well as the NMR structure of ligand-free HCN [12]. Force vectors were chosen in order to widen the binding pocket thereby mimicking dissociation of the ligand.

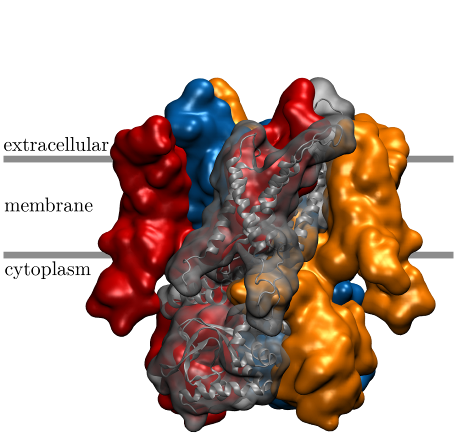

(a) HCN channel tetramer as surface representation colored by chains. The gray subunit in the front is additionally shown as cartoon representation for better orientation.

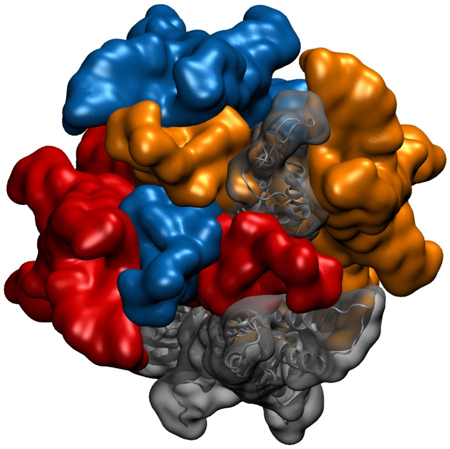

(b) View from the extracellular space onto the top of the channel.

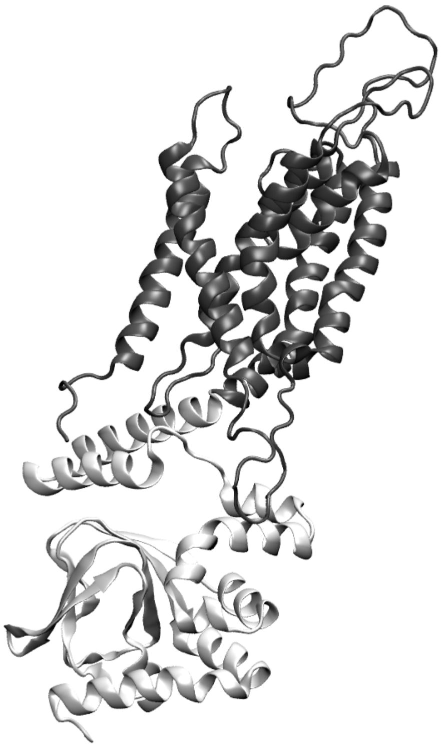

(c) Single subunit of the HCN tetramer with transmembrane domain (gray) and C-terminal domain (white).

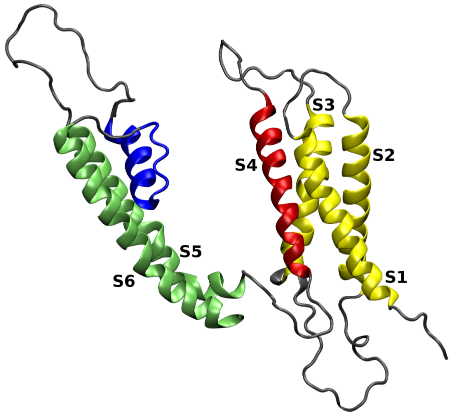

(d) Closeup of the transmembrane domain of one subunit with transmembrane helix S1–S3 (yellow), S4 (red), S5 and S6 (green) and the pore helix and filter region (both blue).

Figure 1: Model of the HCN4 channel. Transmembrane domain is a homology model based on PDB 3LNM. It was joined to the crystal structure of the HCN4 C-terminal region (PDB 3U11).

Figure 2: Error per residue of the HCN4 channel (joined model) visualized as color scale ranging from blue (error < 1.0 Å) via green to red (error > 3.5 Å).

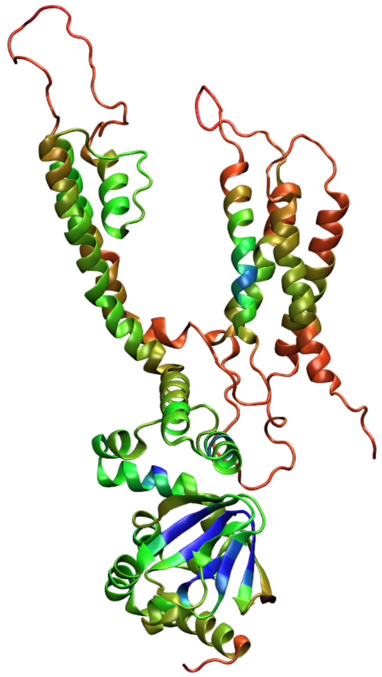

Interaction Cutoff Ikeguchi et al. used a 10 Å cutoff for the interaction network in their LRT experiments [13]. Since other studies using anisotropic network models suggest larger cutoffs [14, 15], the LRT in this thesis was performed under three different cutoff conditions: 10, 13 and 15 Å. Other than requiring a larger force constant to obtain the same amount of distortion, the results for the larger distances (data not shown) did not differ from those of the 10 Å cutoff. Therefore, the subsequent analyses were performed for one cutoff distance only, choosing 10 Å as in the original LRT paper.

Force Application to Single Subunits cAMP binding in HCN channels occurs cooperatively whereby HCN exhibits a dimer of dimers behavior—the probability for one subunit of a dimer to bind a ligand is increased if the other is already occupied [16].

To test whether this cooperativity influences the reaction of the channel in the ligand dissociation model, LRT was performed on only one as well as on all four binding pockets. The displacement of nodes in the subunits without force application was smaller than that of the forced subunit but the general reaction of the channel remained the same regardless of the number of forced subunits. This observation is in line with a study by Benndorf et al. [17] that showed that the binding of the fourth cAMP molecule further contributes to the conformational change.

4 Overlap of $\Delta\boldsymbol{r}$ with Low Frequency Modes

A singular value decomposition was performed on the Hessian matrix to obtain its eigenvalues and -vectors. The overlap $o_i := \Delta\boldsymbol{r} \cdot \boldsymbol{u}_i / (|\Delta\boldsymbol{r}| \cdot |\boldsymbol{u}_i|)$ between the non-degenerate eigenvectors $\boldsymbol{u}_i$ and the displacement vector $\Delta\boldsymbol{r}$ from LRT was computed and is shown in Fig. 4. For identical directions of $\Delta\boldsymbol{r}$ and $\boldsymbol{u}_i$, the respective overlap o_i is 1; if they are orthogonal, it vanishes.

Since the eigenvectors are sorted from low to high according to their eigenvalues, the low frequency modes – those with low corresponding eigenvalues, also called "soft modes" – are on the left side of the plot. The highest overlap of a single mode (no. 8 according to the index in 4) with $\Delta\boldsymbol{r}$ was 0.73 and is shown in 5a. Note that it is irrelevant whether the overlap is positive or negative, since the modes are fluctuations around an equilibrium state [18].

5 Improving the overlap with $\Delta\boldsymbol{r}$

To clarify whether this is the case for cAMP dissociation in our model, linear combinations of the eigenvectors that featured the highest overlap with $\Delta\boldsymbol{r}$ were computed.

We show in Fig. 5b the overlap of a combination of two vectors with $\Delta\boldsymbol{r}$, while Fig. 5a (the sole eivenvector no. 8) is shown for comparison.

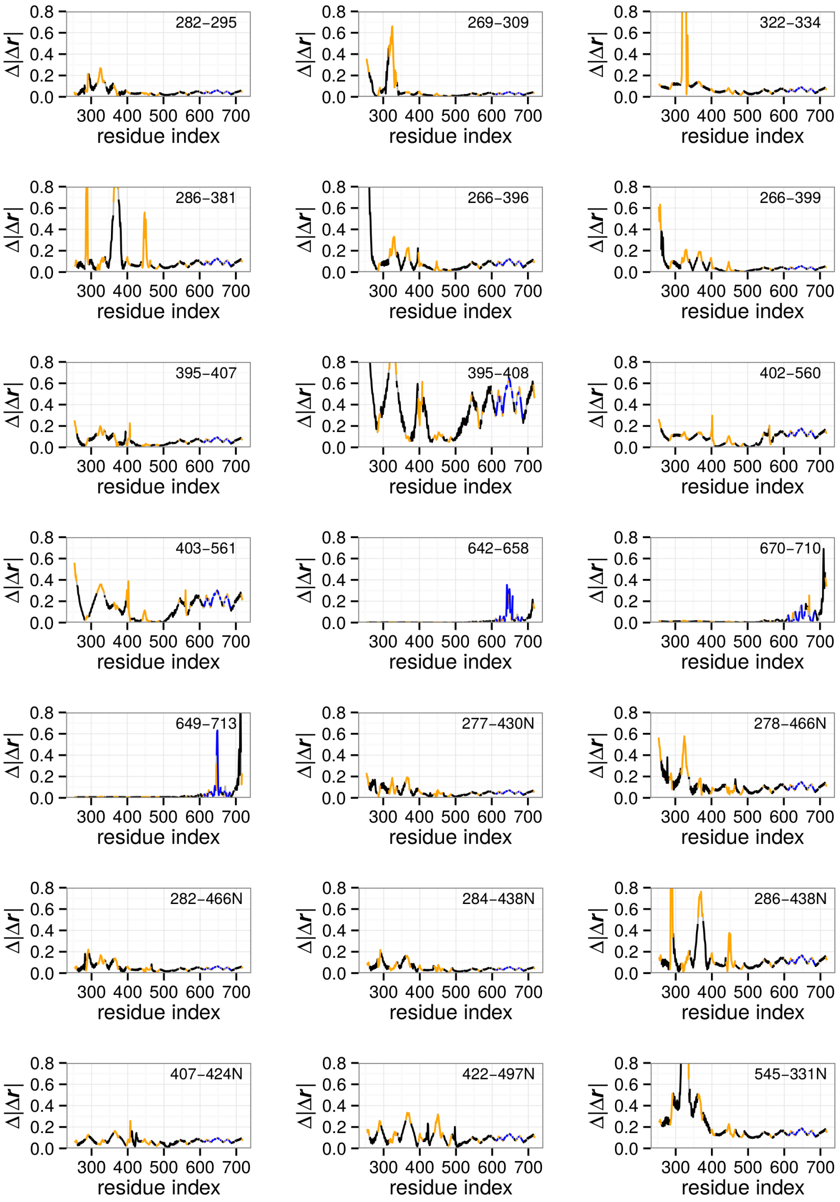

Figure 3: Change of the magnitude of displacement upon force application caused by switching off a contact and its counterparts in all four subunits. The switched off contact is given in the upper right corner of each plot. Residue numbers marked with "N" belong to neighboring subunits in an inter-subunit contact.

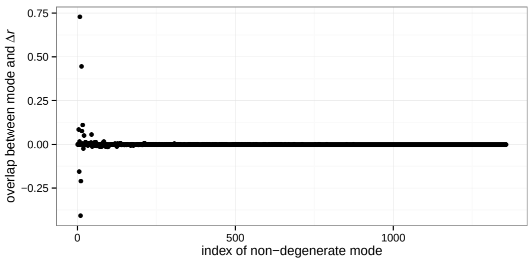

Figure 4: Overlap of 1357 non-degenerate modes with $\Delta\boldsymbol{r}$ from LRT. Modes are sorted according to their corresponding eigenvalues starting with the lowest at index 1.

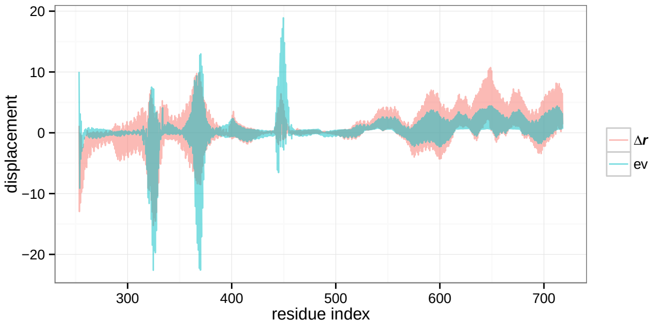

(a) Eigenvector no. 8 (ev, blue) has the highest overlap with $\Delta\boldsymbol{r}$ (red): 0.73.

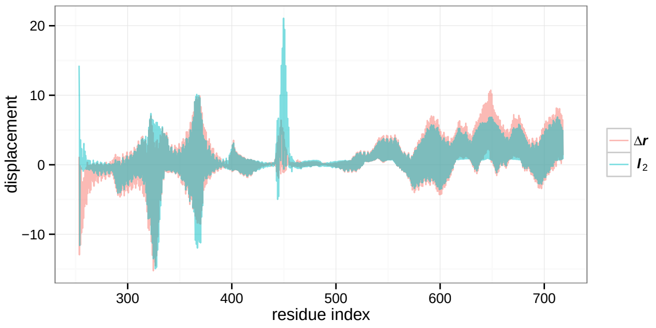

(b) Linear combination of two eigenvectors ($\boldsymbol{l}_2$, blue) with the highest overlap with $\Delta\boldsymbol{r}$ (red): no. 8 and no. 13 (overlap: 0.85).

Figure 5: $\Delta\boldsymbol{r}$ from LRT compared to eigenvectors derived from a singular value decomposition of the Hessian matrix of the system. Plotted vectors were scaled to the same magnitude for better comparability.

6 Effect of Voltage – Synergies to cAMP binding

We mimiced the effect of a cross-membrane potential by applying a force parallel to the z-axis – thus perpendicular to the membrane. We sued two different scenarios: 1) all charged residues were taken into account (AC) and 2) only those charged residues that reside within in transmembrane regions (TM) on the basis that typically the voltage sensor of voltage gated channels is also located in the transmembrane region.

We then computed the shift $\Delta\boldsymbol{r}$ within the LRT approximation upon applying this force. We found only minuscule differences for the two $\Delta\boldsymbol{r}$. For the TM scenario there are 40 basic and 31 acidic residues per monomer in total.

We quantified the similarity of the effect of cAMP binding onto the structural change under LRT to the AC and TM scenario by computing the overlap distance as in Eq. (4) in the main manuscript. We express the distance by the angles $\phi_{\mathrm{cAMP,AC}}$ and $\phi_{\mathrm{cAMP,TM}}$. A smaller value corresponds to better agreement.

Now, this value in itself is hard to assess. To statistically quantify whether the voltage induced, mechanical "reaction" of the channel tetramer is small or large we derived a reference model. The rationale is that the response to voltage is characteristic and fits into the observed synergy between cAMP binding and voltage for channel opening. Therefore, we wanted to know what would be the structural change $\Delta\boldsymbol{r}$ when the same electrical field is present while the (same) partial charges a randomly distributed in the monomers rather than in the wild-type fold.

To this end, we repeated the computation of $\phi_{\mathrm{cAMP},i}$ where i counts the individual randomization experiments. We performed $1,000$ of them.

The overall results are presented in Fig. 6. Clearly, our AC and TM results deviate noticeable from the ones of the reference model. We thus conclude that the response of the channel to a present electrical field along the z-axis agrees quiet well with the effect of cAMP binding.

7 Software

Protein images were rendered with `VMD` [19]. Plots were created in `R` [20] using the `ggplot2` library [21].

References

[1] Arnold K, Bordoli L, Kopp J, Schwede T (2006) The SWISS-MODEL workspace: a web-based environment for protein structure homology modelling. *Bioinformatics* 22(2):195–201.

Figure 6: Overlap angles for randomized charges upon electrical field application; histogram taken over $1,000$ experiments. AC and TM are the two setups described in the text. Clearly, the distribution assigns an empirical p-value of 0 to the thus significant $\Delta\boldsymbol{r}_{\mathrm{AC}}$ and $\Delta\boldsymbol{r}_{\mathrm{TM}}$.

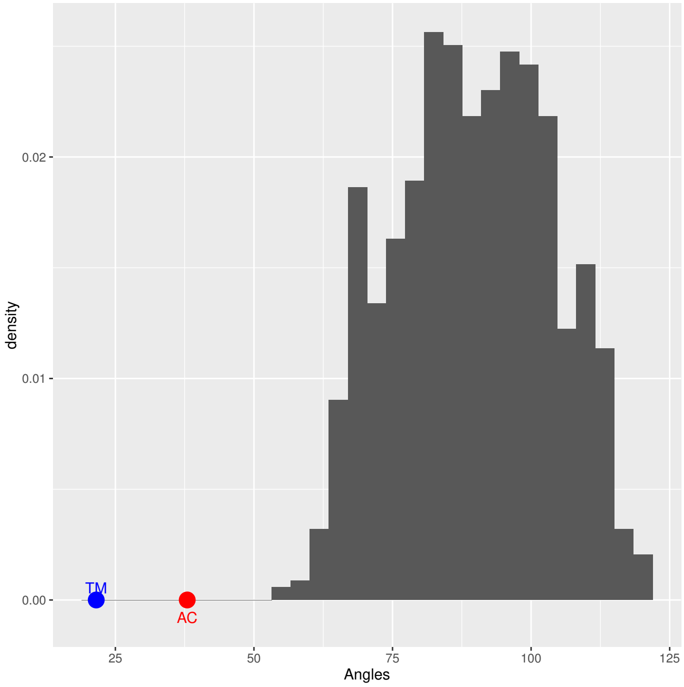

[2] Guex N, Peitsch MC, Schwede T (2009) Automated comparative protein structure modeling with SWISS-MODEL and Swiss-PdbViewer: A historical perspective. *Electrophoresis* 30(S1):S162–S173.

[3] Benson DA, Karsch-Mizrachi I, Lipman DJ, Ostell J, Sayers EW (2010) GenBank. *Nucleic Acids Research* 38:46–51.

[4] Berman HM et al. (2000) The Protein Data Bank. *Nucleic Acids Research* 28:235–242.

[5] Tao X, Lee A, Limapichat W, Dougherty DA, MacKinnon R (2010) A gating charge transfer center in voltage sensors. *Science* 328(5974):67–73.

[6] Šali A, Blundell TL (1993) Comparative protein modelling by satisfaction of spatial restraints. *Journal of Molecular Biology* 234(3):779–815.

[7] Heinig M, Frishman D (2004) STRIDE: a web server for secondary structure assignment from known atomic coordinates of proteins. *Nucleic Acids Research* 32(suppl 2):W500–W502.

[8] Pronk S et al. (2013) GROMACS 4.5: a high-throughput and highly parallel open source molecular simulation toolkit. *Bioinformatics* 29(7):845–854.

[9] Lolicato M et al. (2011) Tetramerization dynamics of C-terminal domain underlies isoform-specific cAMP gating in hyperpolarization-activated cyclic nucleotide-gated channels. *Journal of Biological Chemistry* 286(52):44811–44820.

[10] Benkert P, Künzli M, Schwede T (2009) QMEAN server for protein model quality estimation. *Nucleic Acids Research* 37(suppl 2):W510–W514.

[11] Benkert P, Biasini M, Schwede T (2011) Toward the estimation of the absolute quality of individual protein structure models. *Bioinformatics* 27(3):343–350.

[12] Saponaro A et al. (2014) Structural basis for the mutual antagonism of cAMP and TRIP8b in regulating HCN channel function. *Proceedings of the National Academy of Sciences* 111(40):14577–14582.

[13] Ikeguchi M, Ueno J, Sato M, Kidera A (2005) Protein structural change upon ligand binding: linear response theory. *Physical Review Letters* 94(7):078102.

[14] Atilgan AR et al. (2001) Anisotropy of fluctuation dynamics of proteins with an elastic network model. *Biophysical Journal* 80(1):505–515.

[15] Hamacher K, McCammon JA (2006) Computing the amino acid specificity of fluctuations in biomolecular systems. *Journal of Chemical Theory and Computation* 2(3):873–878.

[16] Kusch J et al. (2012) How subunits cooperate in cAMP-induced activation of homotetrameric HCN2 channels. *Nature Chemical Biology* 8(2):162–169.

[17] Benndorf K, Kusch J, Schulz E (2012) Probability fluxes and transition paths in a Markovian model describing complex subunit cooperativity in HCN2 channels. *PLoS Computational Biology* 8(10):e1002721.

[18] Bahar I (2010) On the functional significance of soft modes predicted by coarse-grained models for membrane proteins. *The Journal of General Physiology* 135(6):563–573.

[19] Humphrey W, Dalke A, Schulten K (1996) VMD: visual molecular dynamics. *Journal of Molecular Graphics* 14(1):33–38.

[20] R Development Core Team (2009) *R: A Language and Environment for Statistical Computing* (R Foundation for Statistical Computing, Vienna, Austria). ISBN 3-900051-07-0.

[21] Wickham H (2009) *ggplot2: elegant graphics for data analysis.* (Springer New York).
